# Early closure of defunctioning stoma increases complications related to stoma closure after concurrent chemoradiotherapy and low anterior resection in patients with rectal cancer

**DOI:** 10.1186/s12957-017-1149-9

**Published:** 2017-04-11

**Authors:** Tzu-Chieh Yin, Hsiang-Lin Tsai, Ping-Fu Yang, Wei-Chih Su, Cheng-Jen Ma, Ching-Wen Huang, Ming-Yii Huang, Chun-Ming Huang, Jaw-Yuan Wang

**Affiliations:** 1 Division of General and Digestive Surgery, Department of Surgery, Kaohsiung Medical University Hospital, Kaohsiung Medical University, Kaohsiung, Taiwan; 2 Department of Surgery, Kaohsiung Municipal Tatung Hospital, Kaohsiung Medical University, Kaohsiung, Taiwan; 3 Division of Colorectal Surgery, Department of Surgery, Kaohsiung Medical University Hospital, Kaohsiung Medical University, No. 100 Tzyou 1st Road, Kaohsiung, 807 Taiwan; 4 Department of Surgery, Faculty of Medicine, College of Medicine, Kaohsiung Medical University, Kaohsiung, Taiwan; 5 Graduate Institute of Clinical Medicine, College of Medicine, Kaohsiung Medical University, Kaohsiung, Taiwan; 6 Department of Radiation Oncology, Kaohsiung Medical University Hospital, Kaohsiung Medical University, Kaohsiung, Taiwan; 7 Department of Radiation Oncology, Faculty of Medicine, College of Medicine, Kaohsiung Medical University, Kaohsiung, Taiwan; 8 Center for Biomarkers and Biotech Drugs, Kaohsiung Medical University, Kaohsiung, Taiwan; 9 Research Center for Environmental Medicine, Kaohsiung Medical University, Kaohsiung, Taiwan; 10 Research Center for Natural Products and Drug Development, Kaohsiung Medical University, Kaohsiung, Taiwan

**Keywords:** Rectal cancer, Concurrent chemoradiotherapy, Defunctioning ileostomy, Defunctioning colostomy, Low anterior resection, Early closure of a stoma

## Abstract

**Background:**

After a low anterior resection, creating a defunctioning stoma is vital for securing the anastomosis in low-lying rectal cancer patients receiving concurrent chemoradiotherapy. Although it decreases the complication and reoperation rates associated with anastomotic leakage, the complications that arise before and after stoma closure should be carefully evaluated and managed.

**Methods:**

This study enrolled 95 rectal cancer patients who received neoadjuvant concurrent chemoradiotherapy and low anterior resection with anastomosis of the bowel between July 2010 and November 2012. A defunctioning stoma was created in 63 patients during low anterior resection and in another three patients after anastomotic leakage.

**Results:**

The total complication rate from stoma creation to closure was 36.4%. Ileostomy led to greater renal insufficiency than colostomy did and significantly increased the readmission rate (all *p* < 0.05). The complication rate related to stoma closure was 36.0%. Patients with ileostomy had an increased risk of developing complications (*p* = 0.017), and early closure of the defunctioning stoma yielded a higher incidence of morbidity (*p* = 0.006). Multivariate analysis revealed that a time to closure of ≤109 days was an independent risk factor for developing complications (*p* = 0.007).

**Conclusions:**

The optimal timing of stoma reversal is at least 109 days after stoma construction in rectal cancer patients receiving concurrent chemoradiotherapy and low anterior resection.

## Background

Fecal diversion is a common technique for managing various surgical situations including congenital diseases, acute or chronic inflammation, acute or chronic colonic obstruction, and malignancy. Two common procedures for fecal diversion are loop transverse colostomy and loop ileostomy. Temporary defunctioning stomas are often created to protect the distal anastomosis in cases where anastomotic leakage may occur. In low-lying rectal cancer patients receiving concurrent chemoradiotherapy (CCRT), the anastomosis can be protected by creating a defunctioning stoma after a low anterior resection (LAR) or coloanal anastomosis (CAA) [1]. However, the complications associated with stool diversion and the morbidity after stoma closure, which may be as high as 47.6 and 34% in reviewed literatures [2, 3], should be considered when constructing a defunctioning stoma. Although the creation of a proper loop transverse colostomy is technically challenging when the splenic flexure is mobilized to allow for a tension-free colorectal anastomosis, opinions vary regarding the choice of ileostomy or colostomy for diverging the stool stream from the perspective of complications before and after stoma reversal. Furthermore, the optimal timing of defunctioning stoma closure ranges widely and is not yet clearly defined in literatures.

## Methods

**Fig. 1:**
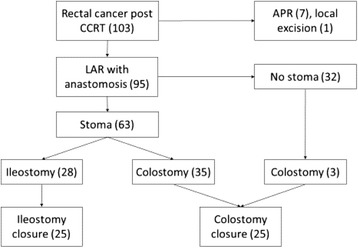
Flowchart of data collection

In our study, data from a single medical center were retrospectively retrieved. This study enrolled 103 patients with rectal cancer with or without distant metastasis who received neoadjuvant CCRT between July 2010 and November 2012. The median distance between the lower margin of the tumor and the anal verge was 4.1 cm (SD = 2.2 cm). Locally advanced disease, metastatic disease at the time of diagnosis, and ultra-low lying early rectal cancer within 5 cm of the anal verge accounted for 55.8, 15.8, and 37.2% of all patients, respectively. In most patients, radiation was through 6- and 10-MV photons by using a three-field technique (posterior and both laterals), as described previously [4]. Pelvic radiation therapy was delivered at a dose of 45 Gy in 25 fractions over 5 weeks and was followed by a boost dose of 5.4 Gy, which was administered to the primary tumor. The CCRT chemotherapy regimens applied in our patients mainly included oral capecitabine (61.7%), oral tegafur (8.5%), FOLFOX4 (13.8%), and FOLFIRI plus bevacizumab (11.7%) for patients with distant metastasis. Patients underwent surgery at 6–10 weeks after completing CCRT. Thirty-three (34.7%) patients received laparoscopic surgery, which was considered controversial for managing low-lying rectal cancer at the time of data collection [5, 6]. The total mesorectal excision (TME) technique was performed for all patients, and extended visceral resection was performed for patients with clinical T4 cancer. Anal sphincter-sparing surgery was performed when possible. Of the 103 patients, 95 received colorectal anastomosis or CAA (Fig. 1). Seven patients who received abdominoperineal resection and one who received local excision were excluded. A temporary defunctioning stoma was intraoperatively created at the discretion of the surgeon. In 63 (66.3%) patients, a defunctioning stoma was created during LAR for low-lying rectal cancer. The mean distance from distal margin of the cancer to the anal verge was 3.6 cm (SD = 1.8) in patients with a stoma and 4.9 cm (SD = 2.6) in patients without one (*p* = 0.022). Among patients without intraoperative defunctioning stoma creation, three received subsequent transverse colostomy for anastomotic leakage. Finally, 66 patients with stomata were analyzed. These patients were regularly followed up in the outpatient department to monitor the constructional and functional outcome of the stoma during the defunctioning period. An antidiarrheal agent and electrolyte solution was not routinely used except in patients with profound stoma output and symptomatic dehydration. The closure of each type of temporary stoma involved the standard procedure including peristomal incision and excision of both limbs, with a hand-sewn end-to-end anastomosis using continuous locking suture and Lembert suture techniques. All operations were performed by the same surgical team. The subcutaneous placement of a drain tube and intravenous administration of prophylactic antibiotics were performed in all patients to prevent the occurrence of surgical site infections (SSIs). In total, 25 of 28 ileostomies and 25 of 38 transverse colostomies were closed after the distal anastomosis was secured and adjuvant chemotherapy (if necessary) completed. The median time from stoma construction to closure was 129 (range, 58–326) days. All demographic data were recorded, and clinical courses before and after defunctioning stoma reversal were compared.

### Statistical analysis

Qualitative variables are expressed as numbers and percentages and the quantitative variables as the median and SD. Comparisons between groups were performed using the χ^2^ test for categorical variables and Mann–Whitney *U* test for quantitative variables. The *α* error was set at 0.05. Logistic regression analysis was performed to identify risk factors for developing complications, and unadjusted odds ratios [and 95% confidence intervals (CIs)] were calculated for morbidity. The receiver operating curve (ROC) was used to determine the timing (days) of stoma reversal that optimally predicted the development of complications after stoma closure. Statistical analysis was performed using JMP 12.1 software (SAS Institute, Cary, North Carolina) for Windows. A *p* value of <0.05 was considered statistically significant.

## Results

The demographic data of the patient populations who received loop ileostomies or colostomies were compared (Table 1). The total complication rate from stoma creation to closure was 36.4% (24/66 patients). Electrolyte imbalance, which occurred in 30.3% (20/66) of patients, was the leading cause of complications, followed by renal insufficiency in 21.2% (14/66 patients). Hypokalemia and hyponatremia accounted for most of the electrolyte imbalance cases. The total complication rate was significantly higher in patients with ileostomy (50.0%) than in those with colostomy (26.3%) (*p* = 0.048; Table 2). In addition, colostomy yielded a lower readmission rate (5.4%) than ileostomy did (35.7%; *p* = 0.002). Moreover, renal insufficiency developed more often before ileostomy closure than before colostomy closure (42.9 vs. 5.3%; *p* < 0.001). The development of electrolyte imbalance was borderline higher in patients with ileostomy than with colostomy (42.9 vs. 21.1%; *p* = 0.057). Stoma prolapse was noted in three of the 28 ileostomy cases, and no stoma prolapse was noted in any of the 38 colostomy cases (*p* = 0.021).

**Table 1:** Demographic data of the loop colostomy and loop ileostomy groups

Variables	Colostomy	Ileostomy	Total	*p* value
Number (%)	38 (57.6)	28 (42.4)	66	–
Age, years (SD)	63.1 (13.1)	63.8 (12.0)	62.9 (12.1)	0.834
Sex (%)				0.408
Male/female	22/16 (57.9/42.1)	19/9 (67.9/32.1)	41/25 (62.1/37.9)	
BMI (SD)	23.44 (4.26)	23.62 (2.93)	23.34 (3.33)	0.887
Histology (%)				0.650
WD/MD/PD	7/25/3 (20.0/71.4/8.6)	3/21/22 (11.5/80.8/7.7)	10/46/5 (16.4/75.4/8.2)	
ypT (%)				0.811
T0/T1/T2/T3/T4	6/3/10/18/1 (15.8/7.9/26.3/47.4/2.6)	6/3/9/9/1 (21.4/10.7/32.1/32.1/3.6)	12/6/19/27/2 (18.2/9.1/28.8/40.9/3.0)	
ypN (%)				0.270
N0/N1/N2	23/7/8 (60.5/18.4/21.1)	20/6/2 (71.4/21.4/7.1)	43/13/10 (65.2/19.7/15.2)	
Metastatic disease (%)				0.381
M0/M1	31/7 (81.6/18.4)	25/3 (89.3/10.7)	56/10 (84.9/15.2)	
Emergent operation (%)	6 (15.8)	1 (3.6)	7 (10.6)	0.091
Locally advanced disease (%)	21 (55.3)	12 (42.9)	33(50.0)	0.319
Distance from anal verge, cm (SD)	3.41 (1.75)	3.93 (1.86)	4.06 (2.20)	0.259
CEA, ng/mL (SD)	6.35 (10.04)	3.16 (2.33)	4.94 (7.32)	0.087

*BMI* body mass index, *WD* well differentiated, *MD* moderately differentiated, *PD* poorly differentiated, *CEA* carcinoembryonic antigen

**Table 2:** Complications before defunctioning stoma closure

Variables	Colostomy (%)	Ileostomy (%)	Total (%)	*p* value
Number	38 (57.6)	28 (42.4)	66	–
Complications	10 (26.3)	14 (50.0)	24 (36.4)	0.048*
Dermatitis	1 (2.6)	1 (3.6)	2 (3.0)	0.827
Renal insufficiency	2 (5.3)	12 (42.9)	14 (21.2)	<0.001*
Electrolyte imbalance	8 (21.1)	12 (42.9)	20 (30.3)	0.057
Hypernatremia	0	1 (3.6)	1 (1.5)	0.304
Hyponatremia	3 (7.9)	6 (21.4)	9 (13.6)	0.581
Hyperkalemia	0	3 (10.7)	3 (4.5)	0.065
Hypokalemia	6 (15.8)	7 (25.0)	13 (19.7)	0.439
Stoma retraction	0	1 (3.6)	1 (1.5)	0.188
Stoma prolapse	0	3 (10.7)	3 (4.6)	0.021*
Stoma stenosis	0	0	0	–
Parastoma hernia	0	0	0	–
Ileus	1 (2.6)	2 (7.1)	3 (4.6)	0.387
Readmission	2 (5.4)	10 (35.7)	12 (18.5)	0.002*
Not reversal	13 (34.2)	3 (10.7)	16( 24.2)	0.061

*p < 0.05

Among 50 patients with defunctioning stoma closure, the median time of stool diversion was 129 days (range, 58–326 days). Patients with colostomy waited longer than patients with ileostomy did (median, 173 vs. 115 days). The median defecation time was 2.7 and 3.1 days after ileostomy and colostomy reversal, respectively (*p* = 0.41). No differences were observed in the median hospital stay between patients with colostomy and those with ileostomy (10.5 vs. 10.9 days; *p* = 0.76).

During the postoperative period after stoma closure, no mortality was recorded. The total complication rate related to stoma reversal was 36.0% (18/50 patients; Table 3). Overall complications developed more often in patients with ileostomy than in those with colostomy (52.0 vs. 20.0%; *p* = 0.017). Thirteen of the 25 (52%) patients with prior defunctioning ileostomy developed unfavorable events including prolonged ileus (*n* = 8) and superficial/deep SSI (*n* = 6). Most patients who suffered from prolonged ileus experienced relief after conservative treatment, and half of the patients with SSI received a subsequent reoperation and open drainage as proper management of a deep incisional infection. Among patients with colostomy, five (20.0%) developed complications after colostomy closure, including two cases of prolonged ileus, two of deep SSI, and one of anastomotic insufficiency.

**Table 3:** Complication and reoperation rates after defunctioning stoma closure

Variables	Colostomy (%)	Ileostomy (%)	Total (%)	*p* value
Number	25 (50)	25 (50)	50	–
Mortality	0	0	0	–
Complications	5 (20.0)	13 (52.0)	18 (36.0)	0.017*
Ileus	2 (8.0)	8 (32.0)	10 (20.0)	0.029*
Anastomotic insufficiency	1 (4.0)	1 (4.0)	2 (4.0)	1.000
Wound infection	2 (8.0)	6 (24.0)	8 (16.0)	0.116
Incisional hernia	0	0	0	–
Primary anastomotic site leakage	0	1 (4.0)	1 (2.0)	0.236
Re-operation	4 (16.0)	5 (20.0)	9 (18.0)	0.210

*p < 0.05

We identified independent risk factors for complications before stoma closure. Age, sex, body mass index (BMI), locally advanced disease, and metastatic disease all had minor effects on the complication rate. The percentage of ileostomy patients with complications during stool stream diversion was significantly higher than that of patients without complications (50 vs. 26.3%; *p* = 0.048; Table 4). Adjuvant chemotherapy for high-risk patients and patients with lymph node metastasis did not alter the incidence of complications (*p* = 0.454). A longer waiting time before stoma closure did not increase the complication rate during the stool diversion period (*p* = 0.406). Multivariate analysis revealed that the odds ratio for the development of complications was 3.71 times higher in patients with ileostomy than in those with colostomy, and it was the single independent risk factor for developing complications before stoma closure (OR 3.71; 95% CI 1.24–11.94; *p* = 0.019).

**Table 4:** Univariate and multivariate analysis of the risk factors for developing complications before defunctioning stoma closure

Variables	With complications (*n* = 24) (%)	Without complications (*n* = 42) (%)	Univariate analysis *p* value	Logistic multivariate regression analysis OR (95% CI), *p* value
Age (SD)	65.5 (13.5)	62.2 (12.0)	0.319	–
Male (%)	13 (54.2)	28 (66.7)	0.316	–
BMI	22.56 (3.70)	24.21 (3.30)	0.175	–
Metastatic disease (%)	4 (16.7)	6 (14.3)	0.800	–
Locally advanced disease (%)	13 (54.2)	20 (47.6)	0.609	–
Distance from anal verge, cm (SD)	3.85 (1.85)	3.51 (1.78)	0.469	–
Emergent operation (%)	3 (12.5)	4 (9.5)	0.708	–
Stoma (%)			0.048*	
Colostomy	10 (41.7)	28 (66.7)		
Ileostomy	14 (58.3)	14 (33.3)		3.71 (1.24~11.94), 0.019*
Time to reversal, days (SD)	141.8 (67.1)	158.5 (66.0)	–	–
Adjuvant chemotherapy (%)	12 (50.0)	17 (40.5)	0.454	

*BMI* body mass index

*p < 0.05

Furthermore, risk factors for reversal-related morbidity were also analyzed. Age, sex, BMI, locally advanced disease, and metastatic disease did not alter the complication rate during the period following stoma closure. Similarly, complications developed more frequently in patients with ileostomy reversal. After defunctioning stoma closure, patients with ileostomy developed more complications than those with colostomy did (52.0% of ileostomy cases and 20.0% of colostomy cases; *p* = 0.017; Table 5). Adjuvant chemotherapy seemed to have no influence on the incidence of reversal-related morbidity. Moreover, a nonsignificant correlation was observed between complications before stoma closure and those after stoma closure (*p* = 0.483). Notably, the postoperative complication rate was significantly higher when the time to reversal was shorter. An average of 120.9 and 169.1 days passed before defunctioning stoma closure in patients with complications compared with in those without complications (*p* = 0.006).

**Table 5:** Univariate and multivariate analysis of the risk factors for developing complications after defunctioning stoma closure

Variables	With complications (*n* = 18) (%)	Without complications (*n* = 32) (%)	Univariate analysis *p* value	Logistic multivariate regression analysis OR (95% CI), *p* value
Age (SD)	66.1 (14.0)	63.5 (11.1)	0.511	–
Male (%)	12 (66.7)	18 (56.3)	0.468	–
Stoma (%)			0.017*	
Colostomy	5 (20.0)	20 (80.0)		
Ileostomy	13 (52.0)	12 (48.0)		2.68(0.67~10.74), 0.161
Time to reversal, days (SD)	120.9 (45.1)	169.1 (70.1)	0.006*	
Time to reversal ≦109 days (%)	10 (58.8)	7 (41.2)	0.002*	6.31 (1.59~25.01), 0.007*
Prior complications (%)	8 (44.4)	11 (34.4)	0.483	–
Metastatic disease (%)	0	4 (12.5)	0.052	–
Locally advanced disease (%)	9 (50.0)	12 (37.5)	0.391	–
Distance from anal verge, cm (SD)	3.67 (1.82)	3.09 (1.48)	0.256	–
Adjuvant Chemotherapy (%)	5 (27.8)	13 (40.6)	0.359	–

*p < 0.05

The ROC was used to determine the timing of stoma reversal that optimally predicted the development of complications after stoma closure. The area under the curve was 0.75, and a 109-day interval from stoma construction to reversal yielded a sensitivity of 64.7% and a specificity of 87.5%. According to the univariate analysis, the closure of a defunctioning stoma within 109 days was a risk factor for developing postoperative complications related to stoma closure (*p* = 0.002; Table 5). In the multivariate analysis, the odds ratio for developing complications after stoma closure was 6.31 times higher in patients with a time to closure ≤109 days (95% CI 1.59–25.01; *p* = 0.007). However, after the multivariate analysis, ileostomy was not an independent factor for postoperative risk (OR 2.68; 95% CI 0.67–10.74; *p* = 0.161).

According to our data, the time to defunctioning stoma closure is related to several factors. The overall development of complications during the defunctioning period had no impact on the waiting time (*p* = 0.406). However, patients with renal insufficiency had earlier defunctioning stoma closure than those without renal insufficiency did (113.8 vs. 163.5 days; *p* = 0.003; Table 6). In addition, patients with readmissions for complications during the stool diversion period tended to have earlier stoma closure (122.5 vs. 161.0 days; *p* = 0.032). By contrast, patients with ileus waited 36.6 days longer (187.5 vs. 150.9 days; *p* < 0.001), and patients with initial ultra-low lying cancer (anastomotic level ≤5 cm) had stoma reversal 42.6 days later (157.6 vs. 115.0 days; *p* = 0.011) than their respective counterparts.

**Table 6:** Factors associated with the time to reversal of defunctioning stoma

	Time to reversal, days (SD)	*p* value
Age		0.479
<70	157.5 (67.1)	
≧70	143.5 (65.6)	
Sex		0.384
Male	145.1 (55.6)	
Female	163.8 (80.6)	
Metastatic disease		0.653
Yes	162.5 (41.2)	
No	151.5 (68.3)	
Locally advanced disease		0.071
Yes	173.8 (79.6)	
No	136.5 (50.0)	
Distance from anal verge		0.011*
≦5 cm	157.6 (68.6)	
>5 cm	115.0 (25.7)	
Complications during defuntioning period		0.406
Yes	141.8 (67.1)	
No	158.5 (66.0)	
Renal insufficiency during defunctioning period		0.003*
Yes	113.8 (36.3)	
No	163.5 (69.0)	
Electrolyte imbalance during defunctioning period		0.562
Yes	163.6 (73.4)	
No	149.1 (64.7)	
Ileus during defunctioning period		<0.001*
Yes	187.5 (3.5)	
No	150.9 (67.2)	
Readmission during defunctioning period		0.032*
Yes	122.5 (42.1)	
No	161.0 (69.8)	

*p < 0.05

## Discussion

Defunctioning stomata play a crucial role in low-lying rectal cancer surgery. According to previous research [7], they reduce not only symptomatic anastomotic leakage but also the need for urgent reoperation when the anastomosis level is ≤7 cm. A defunctioning stoma diverges the stool stream and protects the distal anastomosis in patients who undergo low-lying rectal cancer surgery, particularly those with locally advanced rectal cancer after CCRT. With the creation of a protective defunctioning stoma, the pooled rate of anastomotic leakage requiring relaparotomy is significantly lower in LAR and ultra-LAR than in anterior resection [8]. Neoadjuvant CCRT followed by TME combined with routine defunctioning stoma construction provides both surgical quality and effective local disease control for low-lying rectal cancers [9].

The type of defunctioning stoma created is mostly determined by the surgeon. When the splenic flexure is appropriately mobilized to allow for a tension-free colorectal anastomosis, the creation of a proper loop transverse colostomy is technically challenging and results in low-quality ostomies of tenuous blood supply. When a complication occurs in the pelvis or when a tumor recurs (leading to a repeat resection), a loop transverse colostomy limits the surgical options for the remaining colonic conduit. However, from the perspective of complications before and after stoma reversal, opinions regarding the choice of ileostomy or colostomy for diverging the stool stream remain divided. Our data show that colostomies were performed more often than ileostomies (57.6 vs. 42.4%) for fecal diversion, possibly because of the higher complication rate associated with ileostomies.

The occurrence of dermatitis and renal insufficiency is significantly higher in patients with loop ileostomy than in those with loop transverse colostomy, and after stoma closure, wound infection occurs significantly more often in loop transverse colostomy [10]. The Cochrane database supports the choice of loop ileostomy for fecal diversion, according to results obtained for the occurrence of postoperative stoma prolapse [11]. Another meta-analysis concluded that loop ileostomy reduces the risk of complications, including prolapse and sepsis, during the construction of the stoma but is associated with a high risk of dehydration and occlusion after stoma closure [12]. Defunctioning ileostomy-related complications during stool diversion include electrolyte and acid–base imbalance caused by an increased ileostomy output [13]. The readmission rate in patients with ileostomy is twice that in patients with colostomy after colorectal surgery [14]. The most common etiologies for readmission include postoperative infection, renal failure, and dehydration. The readmission rate for dehydration or renal failure within 30 days following ileostomy creation ranges from 17 to 36%, and an age of >50 years is an independent predictor of readmission for renal failure [15]. Consistent with our findings, the aforementioned observations indicate that ileostomy more frequently leads to renal insufficiency than colostomy does and significantly increases the readmission rate. A higher incidence of stoma prolapse was also observed in our ileostomy patients.

Another concern is that the consequences of stoma reversal are often underestimated [16]. Defunctioning ileostomy closure is associated with a morbidity rate of 18–40% and complications such as bowel obstruction, wound infection, peritonitis, intra-abdominal abscess, anastomotic leakage, enterocutaneous fistula, and bleeding [17–19]. Among these complications, bowel obstruction and SSI are the most common. These complications are associated with a reoperation rate of 3%–8% [20, 21]. Male sex and SSI after primary surgery are independent risk factors for developing wound infections [22]. One recent study suggested that purse-string skin closure is an effective technique for reducing the incidence of superficial SSI [23]. The higher wound infection and total complication rate after ileostomy closure in our study was due to the inclusion of not only deep but also superficial SSIs. The deep incisional infection rate was similar after two types of stoma reversal (3/25 patients for ileostomy and 2/25 patients for colostomy). The nonclosure of stomata is another concern to be addressed. Anastomotic leakage, fistula, advanced primary disease, local recurrence, and comorbidity have been identified as risk factors for nonreversal ileostomy after sphincter-preserving surgery for rectal cancer [24].

The interval between defunctioning stoma construction and stoma closure after LAR for rectal cancer treatment varies, and the median delay in stoma closure ranges from 5.6 to 10.3 months for ileostomy [18, 19, 25, 26]. Increased postoperative complications are associated with a delay in closure. The optimal period is suggested to be approximately 3–6 months [19]. Waterland et al. reported that a delay of >6 months in the reversal of defunctioning ileostomy increases complications and the length of the postoperative hospital stay [25]. The main causes of the delay are adjuvant chemotherapy, medical illness, and anastomotic leakage. In our study, the median time before stoma closure was 129 days. Differences in the interval did not alter the incidence of complications before stoma reversal, but the early closure of a defunctioning stoma was associated with a higher number of postoperative complications after stoma reversal. It is reasonable for a surgeon to close a defunctioning stoma earlier if the patient has renal insufficiency and is frequently readmitted. However, this might be harmful from the perspective of complications after stoma reversal. In the current study, a time to closure of ≤109 days was an independent risk factor for developing complications, including ileus, anastomotic insufficiency, and SSI, during the postoperative period. The precise cause of this phenomenon was not extensively analyzed, but this phenomenon might be attributable to the intraoperative difficulties in manipulation caused by prior unsolved intraperitoneal adhesions, subsequent excessive tissue trauma, and developing complications.

This study has some limitations, including the retrospective design and relatively small sample. Prospective studies with larger samples must be conducted to verify whether a colostomy is the ideal stoma creation procedure and to provide guidance regarding the effective utilization of defunctioning stomata in managing low-lying rectal cancer after CCRT and LAR.

## Conclusions

We believe that the optimal timing of stoma reversal is at least 109 days after stoma construction in rectal cancer patients receiving neoadjuvant CCRT and LAR. Surgeons should especially show more patience in managing patients who experience impaired renal function and are readmitted for complications during the stool diversion period. The difference in the interval between stoma construction and reversal does not alter the incidence of complications before stoma reversal, but the early closure of a defunctioning stoma increases the complications associated with stoma closure.

## Abbreviations

BMI: Body mass index
CAA: Coloanal anastomosis
CCRT: Concurrent chemoradiotherapy
LAR: Low anterior resection
ROC: Receiver operating curve
SSI: Surgical site infection
TME: Total mesorectal excision
